# The physical and mental impact of surviving sepsis – a qualitative study of experiences and perceptions among a Swedish sample

**DOI:** 10.1186/s13690-021-00585-5

**Published:** 2021-05-01

**Authors:** Sabine Apitzsch, Lotta Larsson, Anna-Karin Larsson, Adam Linder

**Affiliations:** 1 The Emergency Department, Skåne University Hospital, Lund, Sweden; 2 Faculties of humanities and theology, Centre for Languages and Literature, Lund University, Lund, Sweden; 3 Region Skåne, Department of Quality Management and Production, Lund/Malmö, Sweden; 4 Faculty of Medicine, Department of Clinical Sciences Lund, Division of Infection Medicine, Lund University, Lund, Sweden

**Keywords:** Critical illness, Experiences, Health related quality of life, Morbidity, Sepsis, Sequelae

## Abstract

**Background:**

Sepsis is a critical illness with high morbidity and mortality rates. Each year, sepsis affects about 48.9 million people all over the world. This study aims to illuminate how sepsis survivors experience sepsis and the impact of sepsis, as well as the health-related quality of life thereafter.

**Methods:**

An interview study with eight sepsis survivors was carried out in Sweden with an inductive qualitative method. The data were analyzed with content analysis.

**Results:**

Four themes were identified during the analysis; *The experience of health care and being a sepsis patient, New circumstances´ impact on life, Family and social interactions,* and *The psychological impact on life*. The lack of information about how sepsis can impact the survivors’ lives and what to expect can lead to prolonged agony. The long recovery time comes as an unexpected and unpleasant surprise to those affected. Initially, the sepsis survivors are almost euphoric that they have survived, which can later lead to chock and trauma when they realize that they could have died. This insight needs to be processed in order to reach reconciliation with life after sepsis.

**Conclusion:**

Sepsis has a huge impact on both physical and mental aspects of life. Many survivors suffer from persistent residual symptoms of varying degrees, to which they have to adapt. The sepsis survivors need individually adjusted information about the sepsis recovery trajectory, and what to expect during and after the hospital stay.

## Background

Sepsis is a life-threatening condition defined as a dysregulated host immune response to a localized infection. Infections such as pneumonia, wound infections, or urinary tract infections may sometimes trigger the immune system to overreact, leading to damage on vital organs such as the heart, lungs and kidneys [1]. Each year sepsis affects about 48.9 million people all over the world [2], and is also one of the major reasons for admission to hospital and critical care units [3, 4]. Sepsis is a critical illness with high morbidity and mortality rates, leading to death for about 10–20% of those suffering from it [5]. The incidence of sepsis in adults in Sweden is estimated to at least 40,000 people each year [6]. In May 2017, WHO adopted a resolution stating that sepsis is a global health problem [7]. Updated international definitions and new clinical criteria for sepsis were presented for adult patients in February 2016. According to the new Sepsis-3 definition, sepsis is defined as a dysregulated host response to an infection leading to life-threatening organ dysfunction [8, 9]. For each hour that adequate treatment with antibiotics, oxygen and fluid treatment is delayed, mortality increases [10], which has led the research field to focus on early detection and treatment of the infection [8, 9, 11]. As a result, the survival rate after sepsis may have increased during the last few years [12, 13].

According to the WHO definition of quality of life (QoL), good QoL is about how the individual perceives his or her life situation in a broader context and in relation to his or her values, expectations and goals. QoL is also linked to the contextually of culture and norms, relationships and family, as well as physical health and mental state [14]. It is difficult to make a clear distinction between health, QoL and HRQoL, previous literature uses the terms interchangeably [15], hence both of the terms are used in this study.

Sepsis survivors experience a reduced HRQoL and impaired functional status compared to other people in general [16, 17]. In the United States, it’s believed that many sepsis survivors are discharged from the hospital with a new, undefined combination of cognitive impairment and physical disabilities such as sensory and emotional problems, which has an impact on work and challenging activities [18]. This could explain the deterioration of their HRQoL [19, 20]. The specific entirety and manifestation of the complications after discharge from hospital are not fully understood [19]. Compared to the average population, sepsis survivors report lower QoL and are less likely to return to work and other activities after being discharged from the hospital [2, 21].

Sepsis can be compared to other critical illnesses [22], such as a stroke, where research has shown that early treatment, support and rehabilitation has a major impact on how extensive the residual symptoms are after the disease, which in its turn affects daily life and the reduction of QoL [23]. It can be assumed that the same effects of early rehabilitation would occur among severely ill sepsis patients, and that family, relatives and friends have a positive impact on recovery [24].

As the number of sepsis survivors increases, it’s of major importance to gain further knowledge about how these patients experience their HRQoL. Further research is required to investigate sepsis survivors’ HRQoL trajectory over time [19] and what kind of support, care and rehabilitation they could benefit from [24, 25].

The aim of this study was to illuminate how sepsis survivors experience the impact of the sepsis episode and their quality of life there after.

## Methods

### Study design and sample

An inductive qualitative method was chosen. Open interviews with eight sepsis survivors were conducted to illuminate the experiences and perceptions of the participants. The inclusion criteria to participate in the study were that the survivors had to be: between 18 and 65 years old, Swedish speaking, and they had to have had an infection that progressed to sepsis or septic shock, as the severity of the infection is associated with an increased need of healthcare and an increased risk of sequelae. It was also a requirement that the sepsis survivors only had had sepsis once. To be included in the study, 3 months should have passed since the sepsis episode, but not more than 5 years. Patients nursed by the author, who is working at an emergency department in the south of Sweden, were also excluded to avoid research bias.

### Data collection

A consecutive sample was selected and recruited from the “Sepsis Forum”, a closed forum on Facebook, where sepsis survivors and/or their relatives can discuss their situation after sepsis. The administrator of the forum posted information about the study and asked the members to give their approval for the researchers to gain access to the forum. When the ethical approval was obtained, an open request was posted with information on how to contact the author if anyone wanted to participate in the study and those who met the inclusion criteria were contacted by telephone. The participants decided the date and place for the interview. Before the interview, the participants received written information via mail about the aim of the study and that they at any given time could withdraw their involvement, without providing a reason or an explanation. Before each interview they submitted their written informed consent for participation in the study.

All interviews were conducted by the first author and the same questions were asked to all participants. Four of the interviews were held in person and the other four by telephone. The participants came from the middle and the south of Sweden. Initially demographic questions were asked and then one general question:” What does health mean to you”? Follow-up questions were asked, such as whether the participants could specify and/or give more examples. An interview guide (appendix 1), was used to ensure that specific areas that were of interest for the study were discussed.

As previously mentioned, some of the interviews were conducted by telephone. According to Burnard, [26], telephone interviewing can have advantages such as feeling anonymous, which provides a sense of security, and there is no geographical limitation. On the other hand, a disadvantage is that facial expressions and gestures cannot be observed [26].

The interviews were audio recorded with the participants’ consent. The transcribed interviews were coded so that no third party would be able to identify any of the participants in the study. The transcribed interviews were stored in a locked cabinet.

### Data analysis

The data analysis was performed using thematic content analysis inspired by Burnard’s framework [27]. Initially, each interview was listened to several times to get a perception of the content. Thereafter, the interviews were transcribed verbatim and read through thoroughly several times. In close connection with each interview, notes of topics were made. A framework of the initial coding was created to facilitate further data processing. Furthermore, an open coding was performed and put together with phrases that described the content of the text. After reduction of the categories in the initial coding, the final framework was created. Afterwards, the coding with reference to the text material was discussed and then divided into sub-headings, categories and themes, until a satisfactory result was reached. Thereafter, the text describing the content of the categories, was written. The next step was to find a quote that captured the core to confirm the credibility. As always, it is important that the researcher stays true to the participants’ intentions for the purpose of validating data. To increase the validity, the author and co-author (AKL) performed the coding and sub-heading separately, and then compared and discussed the results to reach consensus. To enhance the reliability of data interpretation, the author and co-author (AKL) were involved during the whole analysis process. The two other co-authors provided valuable feedback during the categorization process.

### Ethical considerations

The four ethical principles of the Helsinki Declaration [28] were followed. Ethical approval was obtained (2019–00001) by The Swedish Ethical Review Authority. All participants were provided with oral and written information about the study as well as information about how it’s conducted and that the participants could terminate their participation at their convenience.

## Results

**Fig. 1:**
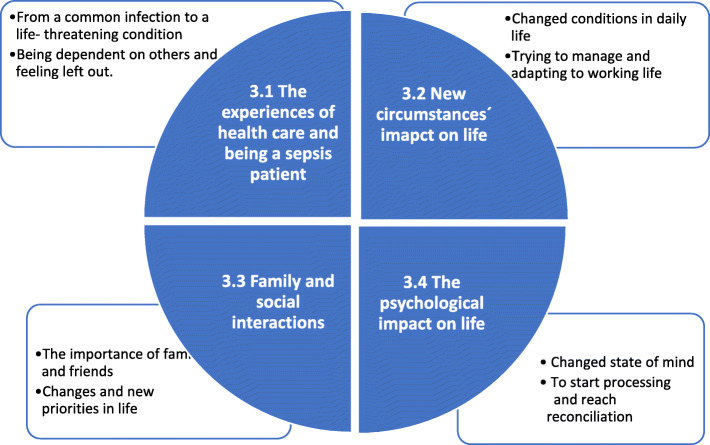
Model over categories. Four main categories emerged after the analysis with two or three subcategories

The interviews lasted from 45 to 80 min. Four of the participants were female and four were male, the age range was from 22 to 58 years, and they lived in different parts of Sweden (Table 2). During the analysis four themes were identified, *The experience of health care and being a sepsis patient, New circumstances’ impact on life, Family and social interactions* and *The psychological impact on life* (see Fig. 1).

**Table 1:** Text units into categories and subcategories

Theme	Category	Sub-heading	Code	Text condensation	Text unit
New circumstances’ impact on life	Changed conditions in daily life	Fatigue	Forget and feeling exhausted	Forget things … and I’m so exhausted because I’m not tired really, I’m tired all day, I can’t sleep at all and I don’t feel like I’m tired from a lack of sleep, just … just exhausted in some strange way.	*”and what I told you about how I lose track of conversations, and how I completely forget things … and I’m so exhausted because I’m not tired really, I don’t know, it’s so strange, I’m tired all day, I can’t sleep at all and I don’t feel like I’m tired from a lack of sleep, just … just exhausted in some strange way.”*

**Table 2:** Demographics of the participants

Age	Gender	Type of interview	Type of infection	ICU	Long-term effects	Comorbiditybefore sepsis	Comorbidity after sepsis	Sepsis episode	Date of interview
22	Female	In person	Thrombo phlebitis	No	Pain, fever, fatigue	Chiari- malformation	Postural tachycardia syndrome (POTS)	January 2019	April 2019
31	Male	In person	Upper respiratory infection	Yes	Cognitive impairment, amputation, wounds, pain, fatigue, pondering	No	No	March 2018	March 2019
38	Female	In person	After childbirth, Sectio	No	Cognitive impairment, impaired short-term memory, fatigue, pondering, depression	No	No	July 2018	March 2019
41	Female	Telephone	Erysipelas	Yes	Frailty of the body, fatigue	No	No	Nov 2017	April 2019
45	Female	Telephone	Pneumonia	Yes	Cognitive impairment, depression, loss self-esteem, fatigue	No	No	Jan 2019	April 2019
51	Male	In person	Influenza	Yes	Cognitive impairment, depression, sleeping problems, loss selfesteem	Pacemaker PM/ICD and Sarcoidosis	No	Jan 2018	April 2019
56	Male	Telephone	Pneumonia	Yes	Total loss of HRQoL, dependent on help with ADL pain, wounds	Diabetes	Myocardial infarction	Dec 2015	April 2019
58	Male	Telephone	Pneumonia	No	Pain, fatigue, sleeping problems	No	No	March 2018	April 2019

The interviews were conducted over 5 weeks between 21/3–24/4 2019. Information on sepsis forum was published in the end of Febrauary 2019, and the first respondent made contact after 2,5 weeks

### The experiences of health care and being a sepsis patient

The participants had difficulties adapting to life both during and after the illness and to managing on their own.

#### From a common infection to a life-threatening condition

Many of the sepsis survivors described that they had minor symptoms of illness such as influenza, pneumonia, or a simple cold before becoming critically ill. *“I had been complaining that I had a sore throat … but one has that every once in a while, during the year and I, or we, had no reason to believe that it was something different this time around” (Interview 1).*

A few of the participants described that they contacted the healthcare for guidance but, were advised to stay at home and see how things developed. When they finally sought care, symptoms had progressed. *“I do not know what happened: if they forgot about me or if they did not take me seriously … I did not get any treatment until seven hours after my arrival … So I had to wait for very many hours and then I just collapsed in the emergency room” (Interview 5).*

#### Being dependent on others and feeling left out

Overall, the sepsis survivors were positive and felt incredibly grateful for the care they received during the hospital stay, but they also feel that improvements are necessary. The importance of positive interactions with the health-care professionals was recurrent in the narratives, because the patients felt dependent on them in every way.” *I told someone [hospital staff] to make sure that the remote control for the bed is nearby … When I woke up … I could not reach the remote control, because … they put it far away from the bed … I tried to call out for help, and I started yelling louder and louder – I just panicked to be honest …” (Interview 3).* To be able to get back to what life had been before the illness, they motivated themselves and began their rehabilitation by setting goals already at the hospital. This was a crucial part of their journey to come home. During the hospital stay, the information provided was scarce and did not include individualized information about what to expect right after discharge or in a long-term perspective. Many meant that this lack of information caused pondering and unnecessary suffering, thus leaving them mentally unprepared. “*… it was more about the information about what’s coming or that it might be this way for a while. Or that it is going to feel this way and that that’s normal …” (Interview 5).* They also felt that an appointment after being discharged would have helped to minimize the sense of feeling left out. Only those who had been admitted to an Intensive Care Unit (ICU) had a follow-up appointment scheduled at the hospital. *“I have not had any follow-up from doctors or nurses, or any follow up regarding the sepsis itself at all …” (Interview 4).*

### New circumstances’ impact on life

All participants talked about how their daily living changed after their sepsis episode and they all referred to various sequalae, such as difficulties with short-term memory and increased sensitivity to stimuli.

#### Changed conditions in daily life

They used the knowledge they already had to make strategies and learned how to deal with everyday life from the mistakes they made. The inner drive and motivation for reaching the goals they set pushed them onwards. They were not prepared for the recovery to take so long and this was perceived as mentally challenging. Fatigue was a major factor affecting this patient group and they described it as complete exhaustion. The fatigue also had negative effects on their sensitivity, ability to focus when reading or conversing and ability to concentrate overall.” *… the brain does not work very well after sepsis … my short term memory is very bad. I forget things very easily …” (Interview 2).*

Almost all sepsis survivors described that they needed support at home and that it took an exceptionally long time until they became independent. *“… I could not handle everything on my own at that point … I could not handle quotidian tasks such as grocery shopping and cleaning …” (Interview 7).* The participants also described difficulties with; pain, loss of appetite, sleeping, anxiety and adapting to the new life conditions. Activities like showering, getting dressed and walking were a struggle.” *I cry from the pain of walking, but still, I think it is so lovely to able to walk …” (Interview 4).* Performing activities that required simultaneous actions, like driving a car, was perceived as challenging. Increased sensitivity to stimuli and impressions, such as light and sound was also a new experience. This sensitivity triggered anxiety, discomfort and stress, and caused nightmares and disturbed sleep. Triggers could be sweating and heavy breathing, which occurred during a sport activity or while watching television, which triggered an underlying memory from the time of the acute illness.

#### Trying to manage and adapting to working life

Many of the participants described difficulties in coming back to their original form. Starting to work again was more difficult than they had expected, and fatigue and forgetting things became an obstacle in this context. Others described losing confidence as they returned to work, despite having many years of experience and knowledge. *“… the first time was the worst of course, because I was all wired up, but I managed to do it and had to take a sedative pill before … I have not felt that way before. Of course, you can be a little nervous, … but I have never been close to a total collapse just because I was going to talk about something that I know so well...” (Interview 3).* After the sepsis episode it was difficult for the participants to get fit and regain their former strength, despite working out. They felt as if the body was not ready for exercise, and not being able to exercise properly was a huge disappointment. A physical sense of frailty persisted for some time and led to temporary removal or complete elimination of activities in their professional lives, but it also affected them at leisure. *“... I cannot be in the frontline; it feels like they can just blow me away like nothing. I don’t dare to” (Interview 7).*

### Family and social interactions

All participants talked about how important family and friends were during the whole process, but also how it can feel to not be able to be the person you once were and that they had new priorities.

#### The importance of family and friends

The sepsis survivors described how happy they were about the support from family and friends. For instance, they delivered food to the hospital if needed, and they were supportive, but they also helped the survivors when health care staff did not respond adequately. Family and friends created a sense of security *“I do not think I was able to identify it. I just knew that I could sleep when my boyfriend was there – then I could relax – but when he was not there I could not sleep. My boyfriend went on sick leave. He was with me all the time” (Interview 7).* All sepsis survivors were able to see that their relationships with family and friends had changed and some even felt closer with their spouses after having sepsis. Many were disturbed by the fact that close relatives had to provide care, but also to what extent they had to do so. They thought too much responsibility was placed on relatives.” *… I do not know what I would have done without my wife. She had to learn how to take care of my wounds and how to help me shower …” (Interview 4). “… I live with my mom and dad, so they had to take a lot of responsibility …” (Interview 6).* The participants also explained that they, to some extent, were different after sepsis and that they now had other priorities, which led to changed relationships with both family and friends.

#### Changes and new priorities in life

The participants found it difficult to relate to their new situation. For most of the survivors the desire to participate in social activities decreased. Partially because of difficulties in staying focused in conversation.*” It was important for me to rest and it was very hard for the people in my surroundings to understand that I wanted to be alone, because I was not that way before the illness … Right after the illness I found it very hard to attend family reunions and such” (Interview 2).* Unfortunately, they perceived others to have difficulties with this change of personality. Being social required a lot of energy, and decreasing social interactions was therefore necessary. This often led to feelings of guilt for not being able to help out at home, attend family gatherings or visit friends. Keeping up the energy that life required was something that persisted, and it had a major impact on the survivors. They thought it was sad to forgo nice activities with family and friends, or to cancel social events. This among other things made it more difficult to see the future in a positive way. This also changed their outlook on life and what they considered to be important, which as mentioned was difficult for family and friends to understand, sometimes leading to irritation for both parties.

### The psychological impact on life

Many described sepsis as a physical illness and was not prepared for the mental part – how to cope and regain their life. They had never heard of or believed that sepsis could have any impact on their mental well-being.

#### Changed state of mind

All sepsis survivors described a lack of patience due to the constant, and sometimes big, setbacks and how difficult they were to overcome. It took very long before they saw any progress, which had a negative impact on their patience. They only knew sepsis as a physical illness and were never given any information about the impact it could have on their mental well-being. The participants said that it took some time until they realized how critically ill they had been, but as their ability to independently perform tasks increased, the memory returned.” *… and I have taken back more of my life than predicted. My wife said that she never thought I would be as well as I am now, but then again it was very bad at first, a year ago I mean” (interview 8).* The trauma of being close to death initially created feelings of gratitude and euphoria, having survived, but later changed to feelings of discomfort and anxiety. Going through sepsis had a huge negative impact on their mental status. All the sepsis survivors described great agony and a lot of pondering on what happened, or what could have happened. After a trauma comes shock and for some this occurred already before being discharged from the hospital, for others at home.” *… since the doctors said that they did not know if I would survive sepsis or not, I even planned my own funeral when they told me …” (Interview 6).*

#### To start processing and reach reconciliation

The trauma was processed in different ways, for example by; writing a diary, reading their hospital records and meeting other survivors – all to understand what they had gone through, but the healing process of the trauma of ‘being close to death’ took very long. Exaggerated thinking about what could have happened was also common. For some of them, there was a huge fear of regaining sepsis and becoming critically ill again. A small symptom they recognized from the time of the illness could create panic and a great sense of fear. They even described being a little hypochondriac. *“I am not a hypochondriac really, but one gets a bit worried. You need to use hand sanitizer all the time and you are more careful. Like, generally you have to be more careful” (Interview 2).* Some wanted to get help from a psychologist connected to the general healthcare services, but it was not easy to get such help. After processing the trauma and reaching a certain acceptance, they were able to accept that they had been critically ill. All participants talked about the tough times they had gone through and about the tremendous efforts required to process that they had been critically ill. Life after discharge was fraught with adversity and trying to get back to normal everyday life, which was a constant struggle. One of the hardest things was to keep up, being hopeful and to look ahead.” *… the psychological part and how it can affect you … I feel that it is a physical illness when I get an infection, when I am in pain and everything hurts … but … it can really mess with your head as well. I was not prepared for that. I was not prepared for sepsis at all, I guess, but I was especially not prepared for the aftermath of it” (Interview 3).*

## Discussion

The sepsis survivors described that the psychological and cognitive impairments with remaining fatigue, lack of concentration, loss of short-term memory, pondering and depression was the worst. Feelings of sadness and depression occurred when the sepsis survivors tried to process the trauma of having been close to death. All of these factors had a negative impact on the daily life and their recovery after returning home.

### Life after sepsis

Many of the participants experienced a depersonalization, almost like they had become a different person in some situations, which had a negative impact on both family and other social relations. The change was due to the need for rest and tranquility. Much responsibility lays with family and relatives after discharge from the hospital, sometimes with shifted roles. For instance, partners tend to become informal caregivers when critically ill patients are released from the hospital and the ICU, especially during the first year, and the same was found in research by Ågård et al. [29]. Most of the sepsis survivors started to work only a few months after discharge, but setbacks were common and took a lot of energy. Some described it as a loss of self-confidence and an overall frailty of the body. It seemed difficult to get back to the professional role at work, mainly due to the psychological and cognitive disabilities, but also due to fatigue. Hence they had new priorities in life. The same findings are described in other studies including people over 65 years of age [20, 30], however many of the sepsis survivors in this study suffered from the same consequences even though the oldest participant was only 58 years old.

Sepsis is comparable to other critical illnesses [22], for example stroke, and according to the recommendations for stroke patients, the general health care services should offer multidisciplinary stroke teams for coordinating discharge and follow-up [23]. Teams like this have a positive impact on the outcome. The sepsis survivors could benefit greatly from a similar program designed for sepsis with a solid plan for their recovery, based on their motivation. It is likely that early interventions could have a positive effect on social ability and physical functionality after sepsis, especially during the first year after hospital discharge [31]..

### Questions need answers

A study shows that it is necessary to feel secure during and after a severe illness, and how trust between healthcare professionals and patients must be established for this feeling to exist [32]. To be able to do so, healthcare professionals should be supportive and responsive to the patients’ needs, and they need to empower them [33]. According to the experiences of sepsis survivors’ loved ones, there is room for improvement regarding care and support during admission as well as after discharge [34]. Some of the participants in this study described that they received some information about what to expect after discharge, but their memories from this period were fragmented. Therefore, the participants had difficulties remembering the information, and sorting out what really had happened, as well as what to expect in the future. It is possible to assume that all the participants received information, at least to a certain extent, but the information was not individually adapted to the sepsis survivors. This probably had an impact on how well the information could be understood, which is an important aspect to consider when giving someone information. How receptive an individual is to information and how well it can be understood plays a major role [35]. Even years after their critical illness, the participants wanted information to fill in the missing gaps. They tried to create meaning from fragmented and scary memories during the recovery, all while wanting to return to a normal life [36]. Previous research shows that during the whole recovery process, patients need a lot of care and support to create existential meaning [37]. Therefore, it’s reasonable to believe that the sepsis survivors would have benefited from an appointment after discharge, with deeper and clearer information about the hospital stay and what to expect further on. Previous research by Reba [33] and Gallop [38], focusing on patients’ experiences 1 year after surviving sepsis, also came to the same conclusion; support as well as a plan for structured follow-up care after discharge is important.

### The psychological impact on life

Individuals exposed to imminent life threats are at high risk of developing mental illness [35]. Life-threatening events such as being critically ill or near death can cause psychological trauma. A study [39] has shown a correlation between the presence of psychological disorders and poor QoL. They also found an association between domains of QoL and various types of psychological complications post-ICU, such as symptoms of post-traumatic stress disorder (PTSD) with anxiety and depression [39]. PTSD is common after critical illness and affects between 10 and 30% of survivors after discharge [40]. The participants in this study also experienced a new sensitivity to various symptoms, such as heavy breathing and sweating, which triggered the underlying memory of being seriously ill. This created feelings of great discomfort. Researchers [19, 20] believe that many who survived sepsis are discharged from the hospital with a new, undefined combination of cognitive impairment and physical disability, which could explain the deterioration in their HRQoL [19, 20]. It’s reasonable to assume that the sepsis survivors at that stage are in some type of crisis. Almost all the sepsis survivors described that it was mentally challenging not to know what to expect after discharge. The fact that it took so long to achieve even the smallest of progress was unexpected and surprising. Previous research found that patients struggled more than 2 years after discharge with existential reconciliation [34]. To prevent psychological problems after discharge, research shows that increased communication during the hospital stay is beneficial [41] and therefore an important issue. The participants in this study experienced that they were left alone to cope with the consequences after returning home. Previous research has focused on critically ill patients post ICU [30, 37, 42–45], however all of the sepsis survivors in this study, post ICU or not, describe similar symptoms of sequelae. In light of the fact that much is unknown about PTSD in connection with the survival of a critical illness, the dynamics of recovering physically and psychologically are important factors to consider when planning the care for the sepsis patients [41, 46]. Many in this study expressed that professional help should have been offered in order to deal with this psychological trauma.

### Study limitations

The analysis reflects findings only from Swedish speaking participants, and therefore no cultural aspects have been considered. Another limitation of this study could be that the participants were recruited from the same closed web forum: the “Sepsis forum”, where all sepsis survivors are willing to share their story. They could therefore be considered as a selected group of outspoken people who dared to share their experience and to make their voice heard. This may have impacted the findings. The exclusion of participants over 65 years may also have had an impact of the findings. Other studies have shown that sepsis survivors over 65 years of age have a very high tendency to get residual symptoms after surviving sepsis [20, 30], for example they tend to need help with activities of daily living (ADL), they can rarely move home again after been discharged from the hospital [20, 30], and those who work are less likely to return to work [19, 39]. Therefore, these participants were not included in this study. The interview time varied from 45 to 80 min, but by using the same thematic interview guide it was ensured that the same topics were addressed in all interviews.

A respondent validation, also called a member check, can strengthen the validity of the study [47], meaning the investigator presents the findings for the informants to achieve agreement. However, this was not done and can be considered as a limitation of this study. Since there is a time-delay between data collection and analysis, there would have been an increased the risk of the informants’ memories differing from the findings.

## Conclusions

Sepsis has a huge impact on all aspects of life, both physical and mental. Many suffer from persistent residual symptoms of varying degrees and varying extent to which they have to adapt. The sepsis survivors need information about what to expect during and after the hospital stay, and said information must be individually adjusted depending on what obstacle they experience. Therefore, a multidisciplinary team around the sepsis survivors are needed for aid with decisions about care, rehabilitation, review of the sepsis episode and follow-up. Further research needs to be done to understand the impact of PTSD post sepsis and what sepsis survivors may need, regardless of admission to intensive care or not, to be able to return to work and a life as normal as possible.

## Data Availability

The datasets used and/or analyzed during the current study are available from the corresponding author on request.

## Abbreviations

WHO: World health organization
QoL: Quality of life
HRQoL: Health-related quality of life
ICU: Intensive care unit
PTSD: Post traumatic stress disorder
ADL: Activities of daily living

## References

[1] Angus D, Van der Poll T (2013). Severe sepsis and septic shock. N Engl J Med.

[2] Rudd KE, Johnson SC, Agesa KM, Shackelford KA, Tsoi D, Rhodes Kievlan D (2020). Global, regional, and national sepsis incidence and mortality, 1990–2017; analysis for the global burden of disease study. Lancet..

[3] Orwelius L, Lobo C, Teixeira Pinto A, Carneiro A, Costa-Pereira A, Granja C (2013). Sepsis patients do not differ in health-related quality of life compared with other ICU patients. Acta Anaesthesiol Scand.

[4] Genga KR, Russel JA (2017). Update of sepsis in the intensive care unit. J Innate Immun.

[5] Stevenson EK, Rubenstein AR, Radin GT, Soylemez Wiener R, Walkey AJ (2014). Two decades of mortality trends among patients with severe sepsis: a comparative meta-analysis. Crit Care Med.

[6] Mellhammar L, Wullt S, Lindberg Å, Lanbeck P, Christensson B, Linder A. Sepsis incindence: a population-based study. OFID. 2016;3(4). 10.1093/ofid/ofw207. 10.1093/ofid/ofw207 PMC5144652 27942538

[7] WHO, World Health Assembly focused on implementation of the International Health Regulations, and improving the prevention, diagnosis and treatment of sepsis, http://www.who.int/en/news-room/detail/26-05-2017-seventieth-world-health-assembly-update-26-may-2017/. Accessed 2 Dec 2018.

[8] Singer M, Deutchman CS, Seymour CW, Shankar-Hari M, Annane D, Bauer M (2016). Third international consensus definitions for sepsis and septic chock (Sepsis-3). JAMA..

[9] Shankar-Hari M, Phillips GS, Levy ML, Seymour CW, Liu VX, Deutschman CS (2016). Developing a new definition and assessing new clinical criteria for septic shock: for the third international consensus definitions for sepsis and septic shock (Sepsis-3). JAMA..

[10] Rhodes A, Evans LE, Alhazzani W, Levy MM, Antonelli M, Ferrer R, et al. Surviving sepsis campaign: international guidelines for management of sepsis and septic shock: 2016. Intensive Care Med. 43(3):377. 10.1007/s00134-017-4683-6. 10.1007/s00134-017-4683-6 28101605

[11] Careprogram on severe sepsis - early idientification and treatment of community-accuired severe sepsis in adults. Region Skåne 2017. http://vardgivare.skane.se/siteassets/1.-vardriktlinjer/regionala-vardprogram%2D%2D-fillistning/sepsis%2D%2D-vardprogram-t-o-m-2019-04-30-rev-171108.pdf; Accessed 3 Aug 2018.

[12] Prescott HC, Costa DK (2018). Improving long-term outcomes after sepsis. Crit Care Clin.

[13] Iwashyma TJ, Cooke C, Wunsch H, Kahn J (2012). J Am Ger.

[14] WHO. Definition of Quality of Life. http://www.who.int/healthinfo/survey/whoqol-qualityoflife/en/. Accessed 12 Dec 2018.

[15] Karimi M, Brazier J. Health, health-related quality of life, and quality of life: what is the difference? Pharmaco Econ. 2016. 10.1007/s40273-016-0389-9. 10.1007/s40273-016-0389-9 26892973

[16] Heyland DK, Hopman W, Coo H, Tranmer J, Mc Coll MA. Long-term health related quality of life in survivors of sepsis. Short form 36: a valid and reliable measure of health-related quality of life. Crit Care Med. 2000. 10.1097/00003246-200011000-00006. 10.1097/00003246-200011000-00006 11098960

[17] Leibovski L. Long-term consequences of severe infections. Clin Microiol Infect. 2013. 10.1111/1469-0691.12160. 10.1111/1469-0691.12160 23397980

[18] Lazosky A, Young BG, Zirul S, Philips R (2010). Quality of life after septic illness. J Crit Care.

[19] Winters BD, Eberlein M, Leung J, Needham DM, Pronovost PJ, Sevransky JE (2010). Long-term mortality and quality of life in sepsis: a systematic review*. Crit Care Med.

[20] Iwashyma TJ, Wesley E, Smith DM, Langa M. Long-term cognitive impairment and functional disability among survivors of severe sepsis. JAMA. 2010. 10.1001/jama.2010.155 Endovascular Aortic Aneurysm Repair—Reply. 10.1001/jama.2010.1553 PMC3345288 20978258

[21] Cuthbertson BH, Elders A, Hall S, Taylor J, MacLennan G, Mackirdy F, et al. Mortality and quality of life in the five years after severe sepsis. Crit Care. 2013. 10.1186/cc12616. 10.1186/cc12616 PMC4057306 23587132

[22] Karlsson S, Ruokonen E, Varpula T, Ala-Kokko T, Pettilä V (2009). Long-term outcome and quality-adjusted life years after severe sepsis. Crit Care Med.

[23] National guidelines for Stroke Care: support for governance and management. 2018 National Board of Health and Welfare. Accessed 12 Dec 2018.

[24] Cuthbertson BH, Rattray J, Campbell MK, Gager M, Roughton S, Smith A, et al. The PRaCtICaL study of nureseled, intensive care follow-up programmes for improving long term outcomes from critical illness: a pragmatic randomised controlled trial. BMJ Open. 2009. 10.1136/bmjb3723. 10.1136/bmj.b3723 PMC2763078 19837741

[25] Scherag A, Hartog CS, Fleishmann C, Ouart D, Hoffmann F, König C (2017). A patient cohort on long-term sequelae of sepsis survivors: study protocol of the mid-German sepsis cohort. BMJ Open.

[26] Burnard P (1993). The telephone interview as a data collection method. Nurse Educ Today.

[27] Burnard P, Gill P, Sterwart K, Treasure E, Chadwick B (2008). Analysing and presenting qualitative data. Br Dent J.

[28] Ethical principles for medical research involving human subjects. World Medical Association Declaration of Helsinki. 1964. http://www.wma.net/policies-post/wma-declaration-of-helsinki-ethical-principles-for-medical-research-involving-human-subjects. Accessed 2 Aug 2018.

[29] Ågård AS, Egerod I, Tönnesen E, Lomborg K (2015). From spouse to caregiver and back: a grounded theory study of post-intensive care unit spousal caregiving. J Adv Nurs.

[30] Hofhuis JG, Spronk PE, van Stel HF, Schrijvers AJ, Rommes JH, Bakker J (2008). The impact of severe sepsis on health- related quality of life: a long-term follow-up study. Anesth Analg.

[31] Gerth AMJ, Hatch RA, Young JD, Watkinson PJ (2019). Changes in health-related quality of life after discharge from an intensive care unit: a systematic review. Anaesth.

[32] Lugton J (1997). The nature of social support as experiences by women treated for breast cancer. J Adv Nurs.

[33] Umberger RA, Todt K, Talbott E, Sparks L, Thomas SP (2021). Advocating for a loved one in the setting of uncertainty: a mixed-methods study among caregivers of sepsis survivors at the point of a sepsis readmission. Dimens Crit Care Nurs.

[34] Umberger RA, Thomas SP (2019). Survivor but not fully recovered: the lived experience after 1 year of surviving sepsis. Dimens Crit Care Nurs.

[35] Cullberg J (2006). Crisis and develpment. Includning trauma psychiatry and late stress reactions. 5th ed.

[36] Ågård AS, Egerod I, Tönnesen E, Lomborg K (2012). Struggling for independence: a grounded theory study on convalescence of ICU survivors 12 months post ICU discharge. Intensive Crit Care Nurs.

[37] Palesjö C, Nordgren L, Asp M (2015). Being in a critical illness-recovery: a phenomenological hermaneutical study. J Clin Nurs.

[38] Gallop KH, Kerr CEP, Nixon A, Verdian L, Barney JB, Beale RJ (2015). A qualitative investigation of patients’ and caregivers’ experiences of severe sepsis. Crit Care Med.

[39] Da Costa JB, Taba S, Scherer JR, Oliveira LLF, Luzzi KCB, Gund DP (2019). Psychological disorders in post-ICU survivors and impairment in quality of life.

[40] Jackson JC, Jutte JE, Hunter CH, Ciccolella N, Warrington H, Sevin C, et al. Posttraumatic stress disorder (PTSD) after critical illness: a conceptual review of distinct clinical issues and their implications. Rehabil Psychol. 2016. 10.1037/rep0000085. 10.1037/rep0000085 27196856

[41] Johnston LB (2014). Surviving critical illness: new insights from mixed-methods research. Smith Coll Stud Soc Work.

[42] Nessler N, De Fontaine A, Launey Y, Morcet J, Mallédant Y, Seguin P (2013). Long-term mortality and quality of life after septic shock: a follow-up observational study. Intensive Care Med.

[43] Corner EJ, Murray EJ, Brett SJ (2019). Qualitative, grounded theory exploration of patients' experience of early mobilisation, rehabilitation and recovery after critical illness. BMJ Open.

[44] König C, Matt B, Kortgen A, Turnbull AE, Hartog CS (2019). What matters most to sepsis survivors: a qualitative analysis to identify specific health-related quality of life domains. Qual Life Res.

[45] Jackson JC, Hart RP, Gordon SM, Hopkins RO, Girard TD, Ely WE (2007). Post-traumatic stress disorder and post-traumatic stressymptoms following critical illness in medical intensive care unit patients: assessing the magnitude of the problem. Crit Care.

[46] Cutler LR, Hayter M, Ryan TA. Critical review and synthesis of qualitative research on patient experiences of critical illness. Intensive Crit Care Nurs. 2013. 10.1016/j.iccn.2012.12.001. 10.1016/j.iccn.2012.12.001 23312486

[47] Burnard P (1991). A method of analyzing interview transcripts in qualitative research. Nurse Educ Today.

